# Lived experiences of adolescents and young adults receiving inpatient psychiatric care

**DOI:** 10.1007/s44192-025-00279-2

**Published:** 2025-11-25

**Authors:** Aaron Nwedu, Jude Ominyi, David Agom

**Affiliations:** 1 David Umahi Federal University of Health Sciences, Uburu, Ebonyi State Nigeria; 2 School of Nursing, Midwifery and Public Health, University of Suffolk, Ipswich, UK; 3 Faculty of Health and Society, University of Northampton, Northampton, UK

**Keywords:** Adolescents, Young adults, Mental health, Psychiatric inpatient care, Coping strategies, Stigma, Nigeria

## Abstract

**Background:**

Adolescents and young adults in low-and middle-income countries (LMICs), face multiple barriers to accessing quality mental health care, shaped by cultural expectations, systemic gaps, and widespread stigma. In Nigeria, little is known about how these factors intersect within psychiatric inpatient settings. This study explored the lived experiences of adolescents and young adults receiving inpatient psychiatric care to inform more culturally responsive, family-engaged, and psychologically supportive models of care.

**Methods:**

Using an Interpretative Phenomenological Approach, we conducted in-depth semi-structured interviews with 28 participants aged 13 to 28 years, all receiving care at a public psychiatric hospital in South-Eastern Nigeria. Participants were purposively sampled and represented a range of diagnostic categories. The hospital, serving a diverse catchment area, reflects a hybrid system of biomedical and traditional models of care. Interviews were conducted over nine months and analysed through iterative, reflexive engagement, supported by member checking to enhance credibility and contextual grounding.

**Results:**

Participants described a range of emotionally charged experiences, with stigma emerging as a major theme. Several reported social withdrawal, strained family dynamics, and shifts in how they were perceived by others. Relationships with peers in hospital varied, offering both connection and tension. Personal coping strategies, such as prayer, art, music, and peer dialogue were commonly described. Despite these efforts, many expressed a desire for consistent psychological support and greater engagement from staff. Hospital environments were viewed as both protective and limiting, with concerns raised about overcrowding, lack of privacy, and emotional neglect.

**Conclusion:**

While the findings cannot be generalised, they offer important insights into the mental health journeys of young people within a Nigerian inpatient setting. Participants called for greater emotional and relational support, staff communication, and culturally sensitive care. These perspectives highlight areas for further research and service improvement in youth mental health provision across similar contexts.

## Introduction

Adolescents and young adults, although sometimes grouped together in mental health research, represent distinct developmental categories [1]. Adolescents are typically defined as individuals aged 10 to 19 years, undergoing rapid biological and psychosocial transitions that influence identity formation, autonomy, and emotional regulation [1, 2]. Young adults, for the purpose of this study, refers to individuals aged 10 to 28 years, a broader age range that includes late adolescence and early adulthood, during which transitions to adult roles and responsibilities become more pronounced [3, 4]. While 28 may appear beyond the conventional upper limit of youth in some international contexts, it aligns with local clinical realities in Nigeria, where adult and youth patients are often co-managed due to resource constraints. Including this extended age group allowed us to explore a broader range of perspectives across the transitional journey from adolescence to adulthood.

Mental health conditions are highly prevalent across these life stages [5], with major depressive disorder, generalised anxiety disorder, and behavioural disorders being leading contributors to illness and disability among adolescents and young adults globally [6, 7]. Around 14% of adolescents are estimated to live with a mental health condition, with most remaining undiagnosed and untreated, particularly in low- and middle-income countries (LMICs) [8]. A meta-analysis by Polanczyk et al., [9] indicated that nearly one in five adolescents worldwide meet diagnostic criteria for a mental disorder, with elevated unmet need reported across many LMICs due to structural limitations, stigma, and cultural dissonance with biomedical models.

Adolescents and young adults across LMICs frequently face barriers to mental health care [10]. These include poor service infrastructure, workforce shortages, limited specialist availability, and minimal integration of mental health into adolescent health systems [10, 11]. Nigeria has fewer than 0.15 psychiatrists per 100,000 people, compared with a global average of 4.5 [12]. Mental health receives limited attention in national funding priorities and remains poorly understood in many communities [13]. Help-seeking is often delayed or avoided due to entrenched beliefs that mental illness stems from spiritual attacks, ancestral punishment, or divine retribution [13, 14].

The sociocultural context of South-Eastern Nigeria, where this study took place, plays an important role in shaping how mental illness is perceived and managed. The region is predominantly inhabited by the Igbo ethnic group, whose moral worldview links well-being with community, family harmony, and spiritual balance [15]. Illness is often explained as a sign of social or moral rupture, spiritual disturbance, or failure to uphold collective obligations [16]. Extended families frequently participate in diagnosis and decision-making, and treatment may begin with traditional healers, churches, or diviners [17]. Public understanding of psychiatric services is influenced not only by these longstanding cultural logics but also by the Igbo diaspora, which introduces new ideas about wellness, education, and modernity [18].

Most research on adolescents and young adults psychiatric care comes from high-income countries. These studies often describe structured therapeutic environments, emotionally supportive staff-patient relationships, and positive impacts from peer support and creative activities [19, 20]. Young adults in these settings report increased self-confidence, improved mood, and a sense of belonging through engagement in recovery-oriented care [21]. Structured psychological interventions and expressive therapies are widely used to support emotional regulation and identity formation [22, 23]. Findings from these high-resource settings do not always align with the lived realities of adolescents and young adults receiving care in Nigeria. Psychiatric inpatient environments are often overcrowded, with limited privacy, minimal staff engagement, and few therapeutic activities [24, 25].

Mental illness remains widely stigmatised in Nigeria, and biomedical care is frequently viewed as secondary to spiritual, moral, or supernatural explanations [13, 14]. For many families, psychiatric hospitalisation is a last resort, often pursued after spiritual or traditional options have been exhausted. Very little research has examined how adolescents and young adults themselves experience inpatient care, particularly how they navigate tensions between biomedical frameworks and traditional belief systems [26]. Within global mental health literature, creative and expressive therapies, including drawing, music, storytelling, and other non-verbal practices have been shown to support emotional regulation and identity work among young people receiving psychiatric care [22, 23]. However, in collectivist societies such as the Igbo context in South-Eastern Nigeria, psychological distress is often understood as a disruption within the family or community rather than as an individualised experience [15, 16].

Moral responsibility for suffering may be shared across generations, and decisions about care are typically made through collective processes involving family elders or spiritual leaders [17]. Approaches that rely heavily on individual introspection or personal expression may not readily align with these social norms. For creative psychological interventions to be effective and ethically grounded in this context, they must be adapted to reflect communal values, spiritual frameworks, and shared modes of meaning-making [27]. Therefore, this study explored the lived experiences of adolescents and young adults to understand how cultural and systemic barriers shape their experiences, and how they find ways to cope within a limited and often fragmented therapeutic environment.

### Aims

This study explored the lived experiences of adolescents and young adults receiving inpatient psychiatric care. Specific objectives include to: (1) understand how young people interpreted and gave meaning to their experiences of mental distress, particularly within a cultural environment where spiritual and moral explanations are often emphasised; (2) explore the strategies they used to manage distress, and how these were influenced by cultural, spiritual, and systemic factors; (3) generate insights that could support the development of care models that are culturally appropriate, emotionally supportive, and inclusive of family and community roles in recovery.

## Materials and methods

### Research design

This study adopted a qualitative design grounded in Interpretative Phenomenological Approach (IPA). This approach draws from phenomenological and hermeneutic traditions, allowing for a detailed exploration of how participants understand and give meaning to their emotional, relational, and cultural worlds [29]. IPA also acknowledges the role of the researcher in making sense of participants’ accounts, a process often described as double interpretation or double hermeneutics [29]. This is particularly relevant in mental health research involving adolescents and young adults, where social context, developmental stage, and cultural values strongly shape both lived experience and its interpretation [29, 30]. This design was selected due to its suitability for research that seeks to understand complex personal and social experiences in under-explored contexts. A phenomenological approach provides a way to centre these voices while attending to the broader cultural and institutional structures that shape their everyday lives [29]. The research was positioned within an interpretive framework that values idiographic depth [29].

### Study setting

The study was conducted at a large, government funded regional mental health hospital located in a peri-urban settlement in South-Eastern Nigeria. This hospital serves as a primary referral centre for individuals across five neighbouring states, with an estimated catchment population of over 20 million people. The facility operates with approximately 300 inpatient beds and offers services for both adult and youth populations. Although there is no dedicated adolescent psychiatric unit, patients aged 13 to 28 are routinely admitted alongside adults due to infrastructural limitations. The hospital functions as a training and teaching centre, hosting student mental health nurses, intern psychologists, and psychiatry residents. The multidisciplinary team includes consultant psychiatrists, clinical psychologists, social workers, occupational therapists, mental health nurses, and general medical officers. Referral pathways include self-referral, family referral, primary healthcare centres, and law enforcement agencies. This setting was selected for its strategic role as a central mental health institution in the region and its diverse service user population, which reflects a broad range of cultural, diagnostic, and socioeconomic backgrounds. These characteristics provided a rich and contextually grounded environment for exploring participants’ lived experiences.

### Participants and recruitment

Twenty-eight participants (16 males and 12 females) aged between 13 and 28 years were recruited through purposive sampling. Inclusion criteria required that participants: (1) had a formal clinical diagnosis of a mental health condition as documented in their medical records; (2) were receiving active treatment either as inpatients or outpatients; (3) demonstrated sufficient insight and communicative ability to provide informed consent; and (4) had the ability to speak and understand English. English proficiency was required to ensure consistency in data collection and analysis. Those who could not communicate in English were excluded. While this limitation is acknowledged, there were no available resources for high-quality interpretation during the study period. Exclusion criteria also included individuals currently undergoing acute crisis intervention, those lacking decisional capacity, or those presenting with ongoing florid psychosis, as determined through multidisciplinary case review.

Clinical stability was assessed collaboratively with the care team, based on observed behaviours, medication compliance, absence of acute symptoms, and the participant’s ability to sustain coherent conversation. Psychiatric diagnoses were confirmed through review of medical records, including case notes and documented assessments from psychiatrists and clinical psychologists. Suitability for participation was determined through a structured eligibility assessment conducted jointly with senior members of the clinical team. To safeguard ethical integrity and address concerns regarding undue influence, gatekeepers such as senior mental health nurses and medical officers introduced the study without pressuring potential participants. Information sheets were provided and explained in plain language. Prospective participants were given between 24 and 72 h to consider participation, consult with their families or carers, and ask questions. Consent was obtained directly from participants by the first author, who was not involved in their care. Where participants were under 18, assent was obtained alongside written consent from a parent or legal guardian. For participants who were unable to write, consent was provided via thumbprint in the presence of an independent witness.

Saturation was monitored during data collection. After 25 interviews, thematic repetition became evident, with no new conceptual insights emerging. Three additional interviews were conducted to ensure depth and confirm saturation, bringing the final total to 28 participants [29, 31]. Table 1 presents demographic and diagnostic details. Conditions are written in full for clarity. Educational background was also recorded to provide additional context regarding social positioning and potential communicative capacity.

**Table 1:** Participant demographic information

Participant ID	Sex	Age (Years)	Diagnosis	Educational Background
P1	Female	13	Generalised Anxiety Disorder	Primary education
P2	Male	13	Major Depressive Disorder	Primary education
P3	Male	14	Cannabis Use Disorder	Primary education
P4	Female	14	Bipolar Affective Disorder	Secondary education
P5	Female	15	Schizophrenia	Secondary education
P6	Male	15	Mixed Mood and Personality Disorder	Primary education
P7	Male	13	Generalised Anxiety Disorder	Primary education
P8	Female	14	Major Depressive Disorder	Secondary education
P9	Female	15	Cannabis Use Disorder	Secondary education
P10	Male	16	Schizophrenia	Secondary education
P11	Male	17	Bipolar Affective Disorder	Secondary education
P12	Female	18	Mixed Mood and Personality Disorder	Secondary education
P13	Male	19	Major Depressive Disorder	Secondary education
P14	Female	20	Social Anxiety Disorder	Secondary education
P15	Male	21	Cannabis Use Disorder	Secondary education
P16	Female	22	Schizophrenia	Secondary education
P17	Male	23	Bipolar Affective Disorder	Secondary education
P18	Female	24	Mixed Mood and Personality Disorder	Secondary education
P19	Male	25	Major Depressive Disorder	Secondary education
P20	Female	26	Generalised Anxiety Disorder	Secondary education
P21	Male	27	Cannabis Use Disorder	Secondary education
P22	Female	28	Schizophrenia	Bachelor of Science
P23	Male	27	Bipolar Affective Disorder	Secondary education
P24	Female	26	Mixed Mood and Personality Disorder	Secondary education
P25	Male	15	Major Depressive Disorder	Secondary education
P26	Female	14	Social Anxiety Disorder	Secondary education
P27	Male	13	Cannabis Use Disorder	Primary education
P28	Female	13	Bipolar Affective Disorder	Primary education

### Data collection

Data collection took place between March and December 2022, allowing sufficient time to engage a diverse group of participants while accommodating the clinical routines of the hospital. All interviews were conducted by the first author, who is an experienced qualitative researcher with a clinical background in adolescent and young adult mental health. This consistent researcher-participant interface supported the development of trust, which is essential for eliciting meaningful and reflective data in studies exploring sensitive experiences [30, 32].

Interviews were conducted in private consultation rooms within the hospital to ensure confidentiality, safety, and emotional comfort. This setting was carefully selected to minimise external distractions and to protect the dignity of participants discussing personal and often distressing experiences [31]. Each session lasted between 60 and 120 min and was audio-recorded with participants’ written or thumb-printed consent. Consent was obtained following a clear and culturally appropriate explanation of the study aims and procedures. For adolescents, parental or guardian consent was secured in advance, while the interviews themselves were conducted privately to respect participants’ autonomy and facilitate openness [32]. A semi-structured interview guide was used to provide consistency while allowing space for participants to direct the conversation. Key domains included understandings of mental illness, perceptions of care, peer relationships, and personal coping strategies. The guide was reviewed by a clinical psychologist and a qualitative research academic to ensure developmental relevance and cultural sensitivity. During data collection, the guide was iteratively refined based on participant feedback and emerging findings, consistent with interpretive research principles [32, 33]. For example, new prompts were added to explore staff communication and participants’ emotional responses to being away from home. These adaptations were made in response to patterns observed during early interviews and reflected the study’s commitment to responsiveness and co-construction of meaning [34].

All interviews were transcribed verbatim by the research team. Transcripts were cross-checked against the recordings for accuracy and then anonymised. Unique participant codes were assigned, and all identifying information was removed. Audio files, transcripts, and field notes were securely stored on encrypted devices and in password-protected files, adhering to institutional data protection standards and ethical guidelines for research with vulnerable populations [35]. A summary of the interview guide and accompanying prompts is presented in Table 2. These prompts were used flexibly, with the researcher following participants’ narratives and probing further where appropriate, in line with the depth-oriented goals of interpretive phenomenological research.

**Table 2:** Interview guide

Primary Question	Associated Prompts
Can you describe your experiences within the mental health hospital?	How would you describe the support you received here? What aspects of the environment were helpful or unhelpful? How did you feel about interactions with staff and peers?
How do you understand and manage your emotional distress?	What emotions or thoughts do you experience? How do you cope when feeling overwhelmed? What role do family, friends, or spiritual beliefs play in how you deal with things?
What social challenges have you faced as a result of your mental health condition?	Have your relationships with others changed? How do people treat you because of your condition? What behaviours or attitudes have been hardest to deal with?
How has the hospital environment affected your well-being?	How do the facilities or routines support or challenge your recovery? What would improve the environment here?
What would you like to see change in mental health care for people your age?	What types of support would make the most difference? What advice would you give to those designing these services?

### Ethical considerations

#### Ethical approval

for this study was granted by the Research Ethics Committee of the Federal Neuropsychiatric Hospital, Enugu on 15 February 2022 (REC/2022/045). All methods were carried out in accordance with relevant guidelines and regulations, including the Declaration of Helsinki and the ethical standards of the Research Ethics Committee of the Federal Neuropsychiatric Hospital, Enugu, Nigeria.

All participants were given clear information about the study’s purpose, what participation involved, and their right to decline or withdraw without consequence. Consent was sought from participants aged 18 and above, while those aged 15 to 17 gave assent alongside guardian consent, consistent with ethical standards for involving minors in health research. Participants were given at least 48 h to consider their decision, and consent was documented either through signature or thumbprint, depending on preference and ability. Confidentiality was maintained throughout. Names were replaced with participant codes, and transcripts were fully anonymised. Interviews were conducted in quiet, private rooms to support participants’ emotional safety. Medical staff were nearby in case participants became distressed, and support or referrals were offered where needed. Interviewers remained sensitive to verbal and non-verbal cues and paused or stopped interviews if discomfort arose. Interviews were conducted in English, the primary language of the hospital and education system, and phrasing was adapted to reflect participants’ social realities. The researcher’s shared cultural background helped to build rapport and encourage openness [33]. Participants’ accounts were represented respectfully and anonymously in dissemination, ensuring their experiences are accurately conveyed while preserving confidentiality. These steps reflect the study’s commitment to ethical research with care, respect, and accountability.

### Data analysis

The analysis followed a structured, yet flexible approach rooted in IPA traditions, enabling a detailed exploration of how participants made sense of their experiences within the psychiatric inpatient setting [36, 37]. Data were analysed iteratively, drawing from participant narratives, field notes, and reflections, with the aim of generating contextually rich, interpretative accounts grounded in participants’ meaning making. Transcripts were read multiple times alongside audio recordings, allowing for close attention to tone, pauses, and emotional intensity [34]. Field notes taken during and immediately after interviews were reviewed to supplement the interpretative process with additional contextual detail [33].

An inductive coding approach was applied during the initial phase. Codes were generated from participants’ own words and phrases, such as “*God is helping me heal*” or “*the noise makes me anxious*”, highlighting key experiences and emotional states [29, 32]. These codes were then examined for patterns of meaning across transcripts and grouped into preliminary categories that reflected both shared experiences and individual divergences. Themes were developed by clustering related codes and exploring underlying meanings. The analytical process involved repeated movement between individual cases and the broader dataset, enabling both idiographic and thematic insights. For instance, codes related to cultural interpretations of illness, spiritual coping, and evolving family roles were synthesised under broader themes like “*Evolving understandings of mental health*” and “*Navigating social relationships*”.

Reflexive dialogue within the research team was maintained throughout. Interpretations were discussed critically in regular team meetings to reduce the influence of individual bias and to enhance interpretive depth. Emerging themes were reviewed alongside original transcripts to ensure that analytic claims remained grounded in the data [38]. The final stage of analysis involved synthesising and presenting the themes using illustrative quotations that preserved participants’ voices and the emotional nuance of their accounts. The narrative structure was carefully constructed to integrate interpretative commentary while foregrounding lived experience [39].

### Ensuring rigour

Several strategies were adopted to enhance the trustworthiness of the study, consistent with established standards for qualitative research [30, 40]. Credibility was achieved through sustained engagement with participants and iterative analysis. The extended data collection period allowed for thoughtful scheduling of interviews and a deeper understanding of participants’ evolving narratives [41]. Dependability was supported through careful documentation of all methodological decisions and analytic steps. A transparent audit trail was maintained, including records of recruitment, interview scheduling, coding iterations, and reflexive memos. This enabled others to understand the logic and flow of the research process [42]. Transferability was strengthened by providing thick description of the setting, participant demographics, and sociocultural context. These detailed accounts enable readers to assess the relevance of findings to similar settings or populations [43]. Participant quotes were integrated throughout the findings to preserve authenticity and allow for contextual interpretation [44]. Confirmability was addressed through multiple strategies, including triangulation of data sources, reflexive journaling, and peer debriefing. Data were drawn from interviews, field notes, and informal observations. Reflexive notes documented the lead researcher’s assumptions, positionality, and responses during the research process [45]. Peer discussions critically interrogated emerging interpretations, helping to safeguard against researcher bias [46]. Authenticity was prioritised by centring participants’ voices and remaining sensitive to both commonalities and divergence in their experiences. The iterative analytical approach facilitated nuanced interpretation while respecting the complexity and individuality of participants’ meaning making [47].

## Findings

Analysis of data resulted in the development of four overarching themes: *evolving understandings of mental health*, *navigating social relationships*, *coping with distress*, and *the therapeutic environment as a mixed experience*. These themes were inductively derived and reflect the dynamic ways in which participants made sense of their experiences over time, influenced by personal reflection, relational encounters, and therapeutic engagement. Each theme is further organised into subthemes and supported by direct quotations from participants to illustrate the analytic claims. Table 3 presents a summary of the themes, subthemes, and illustrative data extracts, providing transparency and grounding the findings in the lived experiences of those involved.

**Table 3:** Themes, subthemes, and illustrative quotes

Themes	Subthemes	Illustrative quotes.
Evolving Understandings of Mental Health	Early confusion and misinterpretation	“curses or punishment” “angered spirits” “unexplained brain issues”
	Gradual clarity through interaction	“doctor explained it’s like a sick brain” “understanding stress as a factor” “not my fault”
Navigating Social Relationships	Isolation and stigma from home	“friends no longer visit” “community views me as cursed” “feeling abandoned”
	Peer relationships within the hospital	“support from peers” “shared understanding” “fights over shared resources”
	Shifting familial roles	“Parents visit weekly” “father avoids contact” “shame from relatives”
Coping with Distress	Spirituality as a foundation	“Prayer helps me calm down” “God gives me hope” “scripture reading for clarity”
	The desire for structured support	“Need for counselling”, “lack of coping skills” “wish for therapy sessions”
	Creative and peer-based strategies	“Writing poems to express emotions” “drawing as a relief” “talking to a roommate”
	Safety and routine	“feeling safe here” “predictable daily routines,“ “shelter from external judgment“

*Themes represent overarching patterns*,* subthemes provide detailed insights*,* and codes include direct examples from participant narratives to illustrate these findings.*

### Evolving Understandings of mental health

Participants’ interpretations of their mental health conditions changed over the course of their stay, influenced by cultural beliefs, personal reflections, and interactions with healthcare providers.

#### Early confusion and misinterpretation

Several participants (11), initially struggled to make sense of their mental health experiences, often drawing on culturally embedded explanations. Spiritual or supernatural interpretations featured prominently in early accounts, with some participants attributing their condition to ancestral displeasure, spiritual attacks, or generational curses. These beliefs were not abstract but deeply rooted in everyday understandings of illness within their communities. Narratives of punishment or moral wrongdoing emerged as common explanatory frameworks, shaping how participants understood both the onset of their distress and the meaning of their hospitalisation. For instance, some believed their illness was caused by curses or spiritual punishments.

“My uncle told me someone must have done something bad to me […] that’s why I started thinking maybe it’s true…” (P2, Female, 14).

“…they say my family angered some spirits, and now this is happening to me. I don’t know if it’s true, but it makes me scared” (P10, Male, 15).

For others, the lack of accessible information about mental health further compounded their confusion. Many participants described being handed medication without a clear explanation of their diagnosis or treatment plan

“I know it’s something about my brain, but I don’t know why it happened or how it can be fixed. No one explains it to me.” (P7, Male, 16).

This initial uncertainty often led to feelings of helplessness and distrust in the treatment process. Participants expressed frustration at being caught between the spiritual explanations provided by their families and the biomedical interventions offered by the hospital.

#### Gradual clarity through interaction

As participants spent more time in the hospital, some began to gain clarity about their conditions. Empathetic conversations with doctors and nurses played a significant role in shifting participants’ understanding from spiritual to medical frameworks. Healthcare providers who used relatable analogies or avoided overly technical language were particularly effective. This is illustrated by the quote below:

“The nurse explained that my brain gets tired just like my body. She said it needs rest and help to recover. That made me feel like it wasn’t something to be ashamed of…” (P12, Female, 17).

However, the transition to a biomedical understanding was not uniform. Many participants continued to grapple with conflicting explanations, especially when their families reinforced spiritual interpretations.

“Even though the doctor said it’s about stress and my brain, my parents still tell me to pray more. It’s hard to believe one thing when everyone at home believes another…” (P6, Female, 15).

The lack of consistent messaging about mental health left some participants feeling caught between two worlds, unsure of which narrative to adopt.

### Navigating social relationships

The interplay of stigma, family dynamics, and peer interactions significantly shaped participants’ social experiences.

#### Isolation and stigma from home

Participants frequently discussed the stigma surrounding mental illness in their communities, describing how it led to feelings of abandonment and isolation. Many participants felt judged by friends and family, who distanced themselves after learning of their hospitalisation.

“I used to have so many friends, but now none of them talk to me. It’s like they’re afraid of me…” (P5, Female, 16).

For younger participants, the rejection from peers was particularly painful, as they longed for social connections but felt constrained by societal attitudes.

“People back home call me names. They say I’m cursed. I try to explain, but they don’t listen…” (P11, Male, 14).

The hospital environment provided some relief from these societal pressures, offering a space where participants felt their condition was normalised.

*“At least here*,* people don’t look at me like I’m crazy. We’re all in the same boat*,* so it feels better.”* (P8, Male, 15).

#### Peer relationships within the hospital

Interactions with peers in the hospital were both supportive and challenging. Many participants found comfort in sharing experiences with others who understood their struggles, fostering a sense of solidarity.

“It’s easier to talk to people here because they know what I’m going through. We help each other when it gets tough.” (P17, Female, 17).

However, overcrowding and competition for resources often led to conflicts, which heightened stress for some participants.

“There are too many of us in one room. We fight over small things, like who gets the best spot to sit. It makes me anxious.” (P13, Male, 16).

Despite these challenges, participants emphasised the importance of peer connections in their recovery journey.

#### Shifting Familial roles

Family dynamics evolved over the course of participants’ treatment. Some (5) participants asserted that family members became more supportive as they began to understand mental illness better.

“At first, my mum didn’t know what to do, but now she comes every week and brings me snacks. It makes me feel loved…” (P6, Female, 15).

Conversely, other participants described feeling abandoned by their families, who distanced themselves due to stigma or a lack of understanding.

“My dad hasn’t come to see me once. I think he’s embarrassed that I’m here.” (P9, Male, 18).

These shifting roles often left participants questioning their place within their families and contributed to feelings of instability.

### Coping within the therapeutic environment

Participants described various coping strategies that were deeply influenced by their cultural beliefs, personal resilience, and critically, the characteristics of the hospital environment itself. While the psychiatric facility offered a degree of safety and stability, systemic challenges such as overcrowding, limited privacy, and mixed interactions with staff also significantly shaped participants’ coping mechanisms and emotional wellbeing.

#### Spirituality as a foundation

For many participants, spirituality was a central pillar of their coping strategies. Prayer, scripture reading, and religious reflection provided comfort, a sense of agency, and hope amidst the uncertainty of their hospitalisation.

“Every night, I pray and ask God to heal me. It gives me hope that one day I’ll feel better.” (P19, Male, 17).

“Reading the Bible calms me when my mind is racing. It reminds me that I’m not alone.” (P4, Female, 14).

The spiritual practices often compensated for the emotional gaps left by an overstretched and occasionally impersonal hospital system. However, participants also recognised the limitations of relying solely on spirituality to address the psychological and emotional complexities they faced.

“Praying helps me feel better, but it doesn’t stop the bad thoughts from coming back. I wish there was something more.” (P20, Male, 15).

Thus, while spirituality remained a vital source of hope, many participants yearned for more tangible, structured forms of psychological support from the therapeutic environment.

#### The desire for structured psychological support

Despite the emotional safety that the hospital environment sometimes provided, participants strongly articulated the need for structured psychological interventions such as counselling, emotional skills training, or therapy groups. Many (9) felt that medication alone was insufficient to address their emotional distress and that the absence of therapeutic support left them feeling isolated during moments of acute need.

“They give us medicine, but that’s not enough. I need someone to teach me how to deal with my feelings.” (P14, Female, 16).

“When I’m upset, I don’t know what to do except sit by myself. I wish someone could show me how to handle it.” (P3, Male, 15).

This unmet need was compounded by the challenges within the therapeutic environment itself, such as the scarcity of trained mental health professionals and the limited availability of private spaces for emotional expression. Overcrowding, in particular, made it difficult for participants to find quiet moments for reflection or to seek help when feeling overwhelmed.

“There are too many people in one room. It’s noisy all the time, and I can’t think or rest properly.” (P13, Male, 16).

“Sometimes I just want to cry, but there’s nowhere to go to be alone. It’s frustrating.” (P2, Female, 15).

Thus, while the hospital aimed to offer stability, its environmental constraints often undermined participants’ coping efforts and highlighted the urgent need for more holistic and developmentally appropriate psychological services.

#### Creative and peer-based strategies amidst environmental challenges

Faced with constraints in the therapeutic environment and limited access to psychological support, participants turned to self-initiated, creative, and peer-supported coping strategies. Activities such as writing, drawing, and poetry emerged as meaningful forms of emotional expression, offering a sense of agency and containment in managing internal distress. These practices provided moments of relief and clarity within the often overstimulating and crowded hospital setting.

“When I feel overwhelmed, I write poems. It helps me let out my feelings without having to talk about them.” (P16, Female, 17).

Peer support also emerged as a vital buffer against environmental stressors. Participants found strength and understanding through relationships with fellow patients, often describing how shared experiences created a sense of solidarity and mutual encouragement even when physical resources were scarce.

“Talking to my roommate helps a lot. He knows exactly how I feel, and we encourage each other.” (P18, Male, 16).

Although these strategies were largely self-initiated, they proved crucial in mitigating some of the distress caused by overcrowding, lack of privacy, and the emotional toll of hospitalisation.

#### Mixed interactions with staff and their impact on coping

Participants’ experiences with hospital staff varied considerably, influencing their emotional coping and overall sense of wellbeing. Positive interactions, characterised by empathy and attentiveness, were critical in reinforcing participants’ emotional resilience.

“There’s one nurse who always checks on me. She makes me feel like I matter.” (P6, Female, 17).

Conversely, negative interactions, such as being dismissed, shouted at, or treated insensitively, often exacerbated participants’ feelings of distress, helplessness, and alienation.

“Some staff just shout at us to be quiet. It makes me feel like we’re not important to them.” (P19, Male, 16).

These experiences significantly shaped participants’ perceptions of the hospital environment as either a source of support or a stressor, further underscoring the pivotal role of staff attitudes and behaviours in facilitating or hindering effective coping.

## Discussion

This study explored the lived experiences of adolescents and young adults receiving inpatient psychiatric care and how they interpret their mental health experiences. A central finding, identified by participants themselves, was the absence of structured psychological interventions within the psychiatric hospital. Participants consistently described receiving pharmacological treatment without parallel opportunities for therapeutic conversation, emotional skills development, or structured support. This echoes previous findings across LMICs, where mental health services are frequently dominated by medication and shaped by limited professional capacity and policy neglect [11–13]. We acknowledge that the categories of ‘adolescents’ and ‘young adults’ are socially constructed and culturally variable. In this study, we retained this distinction to reflect age-specific developmental challenges, while also recognising that clinical practice in Nigeria often does not separate care by these categories. Our inquiry foregrounds the developmental diversity of participants while remaining sensitive to the integrated and resource-limited realities of inpatient care in this context.

Several participants responded to this absence by turning to creative and peer-based strategies. Poetry, drawing, and conversations with fellow patients served as informal means of coping with emotional distress and managing the isolating aspects of hospital life. These findings resonate with existing research in higher-income contexts, where expressive therapies have been used effectively to support adolescents’ and young adults’ emotional regulation and identity work during psychiatric care [19, 21]. However, the significance of these strategies in the Nigerian context must be carefully considered. Many participants continued to draw on spiritual beliefs or viewed their condition through the lens of moral or supernatural causality, shaped by wider family and cultural frameworks [15–17].

The study findings also highlight the tensions that emerge when individualised, biomedical models of care interact with collectivist cultural values. Several participants described how staff explanations about stress and brain function began to shift their understanding of mental illness. Yet others remained conflicted, particularly when family members continued to interpret their experiences through spiritual narratives. These tensions reflect wider findings in global mental health, where misalignment between cultural norms and dominant psychiatric frameworks can create confusion and limit engagement [18, 27].

Rather than viewing creative or psychological interventions as culturally neutral, there is a need to develop approaches that reflect local moral worlds and facilitate collective engagement. Interventions such as group-based expressive therapies, family-inclusive sessions, or narrative practices rooted in communal storytelling traditions may offer more culturally attuned alternatives [13, 22]. Nursing and clinical staff play a crucial role in delivering these forms of support [13], yet their potential is often constrained by limited training and systemic pressures. Investing in culturally relevant psychosocial care, co-produced with adolescents, young adults, and their communities, will be critical for expanding the scope and impact of mental health services in Nigeria.

## Limitations

This study was conducted within a single public psychiatric hospital in South-Eastern Nigeria, and findings reflect the specific sociocultural and institutional context of that setting. Although efforts were made to include a diverse group of participants, the sample may not represent the full spectrum of experiences across different regions, ethnic groups, or care settings. All interviews were conducted in English, which may have excluded some young people who were unable to express themselves fluently in that language. The absence of an adolescent-specific ward also means that participants’ accounts were shaped by exposure to adult-oriented care environments. While these conditions reflect the real-world structure of psychiatric services in Nigeria, they may differ from other national or regional contexts. Researcher positionality may have also influenced the interpretation of participant accounts. Although steps were taken to support reflexivity and minimise bias, meaning making was unavoidably shaped by the interpretative nature of IPA. Findings should therefore be viewed as situated, exploratory insights rather than generalisable claims. Future studies may benefit from including multiple sites, local-language interviewing, and comparative approaches to broaden the understanding of youth experiences in mental health care.

## Implications for practice, policy and future research

Mental health services for young people in Nigeria remain structurally limited, overly reliant on medication, and disconnected from cultural contexts of distress. The findings of this study suggest an urgent need to introduce structured psychological interventions within psychiatric hospitals, delivered alongside medical treatment. Adolescents and young adults are not passive recipients of care; many demonstrated agency in their coping strategies, especially through creative and peer-based methods. Mental health professionals should be supported to develop these into structured therapeutic offerings, such as group art sessions or emotionally focused group counselling. Staff training in culturally sensitive psychological skills, such as narrative therapy, problem-solving, or brief trauma-focused work, may help meet this need.

At the policy level, integrating culturally grounded, developmentally appropriate interventions into national mental health frameworks is essential. Health policies should explicitly support family-inclusive models of care, recognising the collectivist values that shape how mental illness is interpreted and responded to in Igbo society and other Nigerian contexts. Resources should be allocated not only for medication and staffing but also for psychological therapies that align with local values and communication styles. Community engagement is also key. Collaborations with religious leaders, educators, and traditional healers can help promote mental health literacy and reduce stigma while fostering trust in psychiatric services.

Future research should explore the cultural adaptation of psychological interventions for use in inpatient settings. Mixed-methods and co-design studies could assess how expressive or narrative therapies might be integrated into everyday care routines without undermining collective cultural norms. Research should also investigate the long-term outcomes of culturally adapted interventions on emotional recovery, stigma resilience, and social reintegration for young people. Supporting these lines of inquiry will be essential for developing youth mental health services that are both clinically meaningful and culturally relevant.

## Conclusion

This study highlights a clear gap in structured psychological interventions, as identified by participants themselves, and points to the emotional and developmental costs of relying solely on pharmacological care. While many young people turned to informal strategies, spirituality, peer connection, and creative expression, these were not always sufficient to meet their deeper needs for support, understanding, and recovery. Findings reveal the tension between individualised models of therapy and the collectivist values embedded within the Igbo cultural context with participants negotiating between spiritual and biomedical interpretations of their distress. Creative and expressive therapies, while promising, must be adapted to fit cultural values that prioritise community, family engagement, and spiritual meaning.

A culturally grounded, developmentally informed approach to youth mental health care is both necessary and achievable. Interventions must be designed with, rather than for, young people, integrating traditional practices and collective healing methods where appropriate. Strengthening the role of nurses and mental health staff in delivering culturally relevant psychological support could provide a foundation for more compassionate, responsive care. The voices of participants in this study should inform not only clinical practice, but broader health policy and service design. Their accounts point to a future in which mental health care honours both the cultural worlds and emotional needs of the young people it seeks to support.

## Data Availability

The data that support the findings of this study are available from the corresponding author upon reasonable request.

## Abbreviations

IPA: Interpretive phenomenological analysis
LMICs: Low-and Middle-Income Countries
EBP: Evidence Based Practice
WHO: World Health Organisation
CBT: Cognitive Behavioural Therapy
